# ACBP is an appetite stimulator across phylogenetic barriers

**DOI:** 10.15698/cst2020.02.211

**Published:** 2020-01-20

**Authors:** Frank Madeo, Nektarios Tavernarakis, José M. Bravo-San Pedro, Guido Kroemer

**Affiliations:** 1 Institute of Molecular Biosciences, NAWI Graz, University of Graz, Humboldtstrasse 50, 8010 Graz, Austria.; 2 BioTechMed Graz, Austria.; 3 Institute of Molecular Biology and Biotechnology, Foundation for Research and Technology-Hellas, Nikolaou Plastira 100, Heraklion 70013, Crete, Greece.; 4 Department of Basic Sciences, Faculty of Medicine, University of Crete, Heraklion 71110, Crete, Greece.; 5 Metabolomics and Cell Biology Platforms, Gustave Roussy Cancer Campus, Villejuif, France.; 6 Inserm U1138, Centre de Recherche des Cordeliers, Sorbonne. Université, Université de Paris, 15 rue de l'école de médecine 75006, Paris, France.; 7 Team “Metabolism, Cancer & Immunity”, équipe 11 labellisée par la Ligue contre le Cancer, Paris, France.; 8 Pôle de Biologie, Hôpital Européen Georges Pompidou, AP-HP, Paris, France.; 9 Suzhou Institute for Systems Medicine, Chinese Academy of Sciences, Suzhou, China.; 10 Karolinska Institute, Department of Women's and Children's Health, Karolinska University Hospital, Stockholm, Sweden.; # Share senior co-authorship.

**Keywords:** anabolism, anorexia, appetite, catabolism, obesity

Human civilization is unique with respect to the organization of our natural environment, allowing constant supply of nutrients to most individuals in developed countries, contrasting with the natural fluctuation in food sources found in wildlife. Indeed, scarcity of nutrients has been a continuous threat for most living organisms, thus leading to the evolution of sophisticated strategies for coping with dwindling food supply.

In eukaryotic organisms, one of the universal mechanisms of adaptation to nutrient stress is the activation of autophagy, accompanying a switch from anabolic to catabolic conditions. Through the sequestration of portions of the cytoplasm within autophagosomes, followed by their digestion in lysosomes, cells can convert macromolecules into nutrients and building blocks for adaptive stress responses [1, 2].

Following the general rule that intracellular stress is communicated to the extracellular world [3], autophagy leads to the secretion of one particular phylogenetically ancient protein, which is acyl coenzyme A-binding protein (ACBP, also called diazepam-binding inhibitor, DBI) into the extracellular space. This phenomenon has been first documented for unicellular fungi and optionally multicellular slime molds [4], and has later been confirmed for mammalian cells [5].

ACBP/DBI is a small protein (87 amino acids in mammals, with a 58% sequence conservation between humans and the yeast *Saccharomyces cerevisiae*) that has rather distinct functions, depending on its location. As an intracellular protein, it binds acyl coenzyme-A molecules, facilitating their intracellular trafficking. As an extracellular protein, it binds to cell surface receptors to stimulate signals that affect the behavior of cells and organisms in an autocrine, paracrine and endocrine fashion [5, 6].

In *S. cerevisiae*, there is only gene coding for ACBP/DBI (*acb1*). Extracellular yeast ACBP/DBI acts on the pheromone receptor Ste3 to stimulate sporulation. Ste3 is a seventransmembrane G-protein-coupled receptor that responds to the mating-type a-factor pheromone to facilitate cytogamy (cell-to-cell fusion) between haploid gametes during the mating process. In addition, yeast ACBP/DBI stimulates Ste3-dependent sporulation, which may constitute an advantage in conditions of starvation because it allows yeast to move to other food sources [7].

In the nematode *Caenorhabditis elegans*, there are several genes coding for ACBP/DBI orthologs (*acbp-1 to acbp-6*). However, knockdown of one single among these genes (*acbp-1*) is sufficient to profoundly affect the behavior of the animals with respect to food intake. Thus, knockdown of *acbp-1* caused a decrease in pharyngeal pumping coupled to reduce uptake of bacteria into the intestinal tract. This finding suggests again that ACBP/DBI stimulates food intake.

In the fruit fly *Drosophila melanogaster*, a gene called *Anorexia* (*Anox*) codes for an acyl-CoA-binding protein with an ankyrin repeat domain. Flies bearing a defect for this gene, exhibit reduced feeding activity and mouth hook movement, which is the fly equivalent of mastication. Hence, in this species yet another ACBP/DBI analogue might be involved in appetite control [8].

In mice (*Mus musculus*), like in humans, there is only one gene coding for ACBP/DBI. Administration of the recombinant ACBP/DBI protein or its transgenic overexpression in liver cells, causing an increase in ACBP/DBI plasma levels, leads to hyperphagy and triggers lipo-anabolic reactions favoring adiposity, obesity and fatty liver. In sharp contrast, neutralization of ACBP/DBI by injection of antibodies reduces food intake and favors lipocatabolic reactions including triglyceride lipolysis and fatty acid oxidation, thus reducing fat mass [5, 9]. Mice that are rendered obese by a high-fat diet or that become spontaneously obese (on a normal diet) due to a genetic leptin deficiency exhibit elevated ACBP/DBI RNA and protein levels in their tissues, as well as increased ACBP/DBI protein in their blood [5, 9].

In humans (*Homo sapiens*), the body mass index strongly correlates with circulating ACBP/DBI levels. Thus, obesity is coupled to supranormal plasma levels of ACBP/DBI, while anorexia nervosa is accompanied by abnormally low circulating ADBP/DBI concentrations. Dietary interventions causing weight loss cause a transient reduction in ACBP/DBI mRNA expression in the periumbilical fat, while successful bariatric surgery results in reduced ACBP/DBI plasma levels. This suggest a role for ACBP/DBI in the pathogenesis of obesity as well [5].

**Figure 1:**
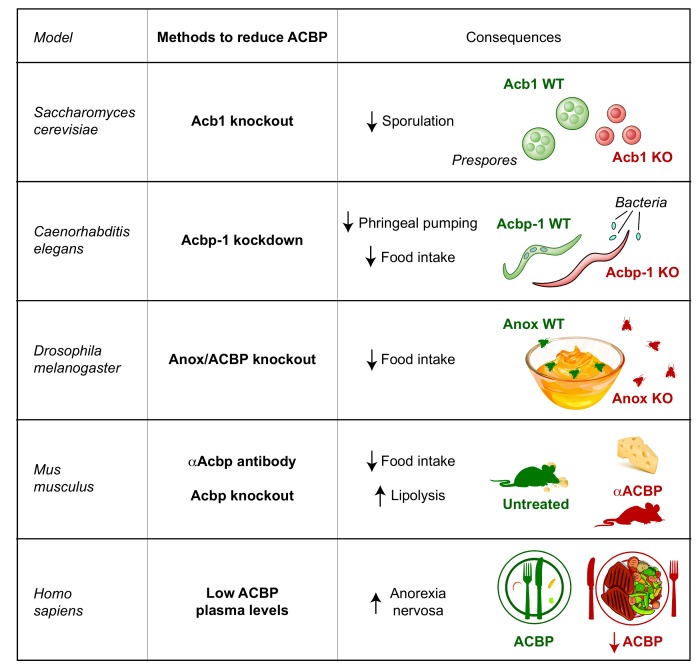
FIGURE 1: Main consequences of neutralization/removal of ACBP/DBI in yeast (*Saccharomyces cerevisiae*), worms (*Caenorhabditis elegans*), flies (*Drosophila melanogaster*), mouse (*Mus musculus*) and human (*Homo sapiens*).

In sum, it appears that ACBP/DBI has an appetite-stimulatory role across phylogeny, from yeast to nematodes, flies, mice and (presumably) humans **(Figure 1)**. That said, there are species specificities, because ACBP/DBI acts on a metabotropic receptor (Ste3) in yeast, but on ionotropic gamma-aminobytyric (GABA) A receptors in mice [7], suggesting that the effector of ACBP/DBI have changed during evolution. Moreover, in yeast it appears that the genetic removal of ACBP/DBI inhibits autophagy, contrasting with findings in *C. elegans*, mice and human cell cultures in which removal ACBP/DBI stimulates autophagy [5, 7]. Whether autophagy modulation is involved in appetite control has not yet been elucidated. It will be important to determine the precise mode of action of ACBP/DBI to understand whether it is possible to target this pathway not only by neutralizing the ligand, but perhaps also by blocking the receptors or post-receptor signal transduction pathways for appetite control.
